# A Prefrontal Neuromodulation Route for Post-Traumatic Olfactory Dysfunction: A Perspective Supported by Recovery During Left-DLPFC rTMS

**DOI:** 10.3390/brainsci16010099

**Published:** 2026-01-17

**Authors:** Chiara Di Fazio, Sara Palermo

**Affiliations:** 1 Department of Psychology, University of Turin, 10124 Turin, Italy; chiara.difazio@unito.it; 2 International School of Advanced Studies, University of Camerino, 62032 Camerino, Italy; 3 Neuroradiology Unit, Diagnostic and Technology Department, Fondazione Istituto di Ricovero e Cura a Carattere Scientifico (IRCCS) Istituto Neurologico Carlo Besta, 20133 Milan, Italy; 4 Neuroscience Institute of Turin (NIT), 10124 Turin, Italy

**Keywords:** post-traumatic olfactory dysfunction, hyposmia, repetitive transcranial magnetic stimulation (rTMS), dorsolateral prefrontal cortex (DLPFC), neuromodulation, olfaction, mild traumatic brain injury (mTBI)

## Abstract

Post-traumatic olfactory dysfunction (PTOD) is a common and often persistent sequela of mild traumatic brain injury (mTBI), with limited evidence-based treatment options. We propose that high-frequency rTMS applied to the left dorsolateral prefrontal cortex (DLPFC) may support olfactory recovery via top-down modulation of distributed olfactory, attentional, and reward networks, and we outline key mechanistic and methodological considerations for future studies. We summarize the case of a 70-year-old woman with severe post-traumatic hyposmia persisting for ~5 months, who underwent a 12-week, 10 Hz rTMS course over left DLPFC (36 sessions; 800 pulses/session). Using a structured door diary and repeated ratings across odour categories, she reported stepwise improvement starting around sessions 10–12 (re-emergence of pungent odours) and progressing to broad restoration, including subtle fragrances, by treatment end; no adverse events occurred. While causality cannot be inferred from a single case, this pattern is consistent with a network-level neuromodulatory effect and motivates controlled trials combining standardized olfactory testing with neurophysiology and neuroimaging.

## 1. Perspective and Rationale

Post-traumatic olfactory dysfunction (PTOD) is a well-recognized consequence of closed-head injury, representing up to 20% of all acquired olfactory disturbances [1,2]. Mechanisms include shearing of olfactory fila at the cribriform plate, microhaemorrhages in the orbitofrontal cortex (OFC), oedema of the olfactory bulb, or diffuse axonal injury disrupting fronto-subcortical networks [3,4,5,6]. Even in the absence of overt lesions on CT, patients frequently experience persistent reduction in olfactory sensitivity or qualitative distortions, with substantial impact on emotional well-being, appetite, social behaviour, and safety [7,8,9].

Spontaneous recovery may occur within the first year but is unpredictable, and no established rehabilitative protocols exist. Olfactory training has shown moderate benefits, though effects are variable and require prolonged engagement [1,6,10].

High-frequency repetitive transcranial magnetic stimulation (rTMS) is an innovative neuromodulatory approach increasingly used to enhance neuroplasticity and functional connectivity in frontal networks [1,8,11,12,13,14,15,16]. Neuroimaging and electrophysiological studies demonstrate that rTMS targeting the dorsolateral prefrontal cortex (DLPFC) can induce immediate and sustained changes in cortical excitability, with effects extending to regions such as the insula, thalamus, and reward circuits [15,16,17]. These network-level modulations have been hypothesized to play a role in alleviating cognitive and affective symptoms present in various neuropsychiatric disorders [17,18,19,20]. The DLPFC is heavily interconnected with subcortical areas such as ventral striatum/nucleus accumbens, which play a vital role in processing reward [21,22,23]. Stimulation of the DLPFC has been shown to increase dopamine activity between these interconnected regions, suggesting a mechanism by which rTMS may impact mood and sensory integration, including olfactory function, along with other senses [17,18,24,25,26,27,28,29]. This network-based perspective supports the rationale for targeting the DLPFC in interventions aimed at enhancing neuroplasticity and functional recovery after brain injury [1,2,8,14,16,25,30,31,32,33,34,35,36,37,38,39,40].

Recent network-based models suggest that prefrontal neuromodulation may influence multisensory processing by enhancing top–down attentional gain, facilitating signal-to-noise ratios, and promoting reorganization of partially impaired sensory circuits [1,2,6,8,41]. For example, pairing rTMS with pleasant olfactory stimulation or other reward-based tasks has been shown to enhance activation of the dopaminergic reward system and may yield greater clinical benefits than rTMS alone [1,42,43,44,45]. Such multimodal approaches are being explored in the treatment of depression and other neuropsychiatric disorders, and they offer a promising avenue for addressing post-traumatic olfactory dysfunction (PTOD), where traditional rehabilitative options remain limited [22,29,46]. However, empirical evidence on rTMS for olfactory dysfunction remains scarce.

In this Perspective, we outline a network-based rationale for considering prefrontal rTMS as a candidate, top-down intervention for PTOD, and we use an illustrative clinical vignette from our neuromodulation program to support the plausibility of this approach and to highlight priorities for future research.

### 1.1. Evidence Snapshot: Established and Emerging Interventions for Acquired Olfactory Dysfunction

**Figure 1:**
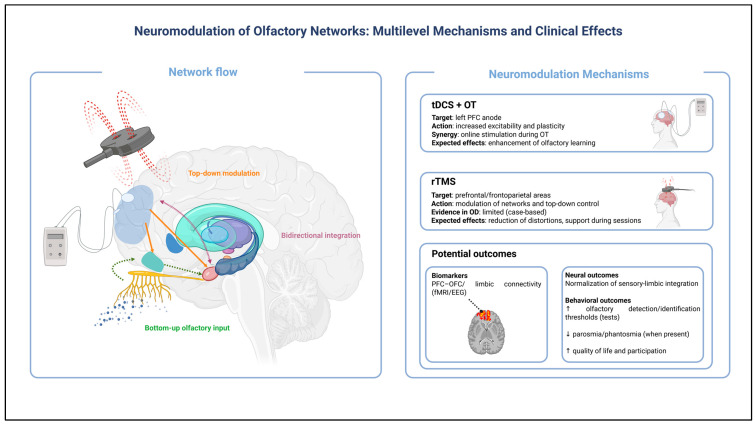
Multilevel neuromodulation of olfactory networks and putative clinical effects.

Therapeutic approaches for acquired olfactory dysfunction span evidence-based rehabilitation strategies and a growing set of emerging interventions (Table 1). Olfactory training remains the most consistently supported first-line option, but recent advances suggest a broader—and potentially synergistic—treatment landscape. In particular, biologic approaches and neuromodulation are gaining attention because they may engage mechanisms that training alone may not fully address, including network-level excitability, top–down modulation of attention and reward, and the plasticity of olfactory–limbic circuits (Figure 1). Although these approaches still require protocol harmonization and stronger confirmatory evidence, they offer a compelling route toward mechanism-informed and personalized care, especially for persistent or treatment-resistant cases.

The figure illustrates the integration of bottom–up olfactory inputs with top–down prefrontal modulation and bidirectional interactions within olfactory–limbic circuits. Neuromodulatory approaches, including transcranial direct current stimulation (tDCS) combined with olfactory training and repetitive transcranial magnetic stimulation (rTMS), are shown as targeting prefrontal and frontoparietal regions to modulate network excitability, plasticity, and top–down control. Potential outcomes span neural and behavioral levels, including normalization of sensory–limbic integration, changes in prefrontal–olfactory connectivity (as assessed by EEG/fMRI), improvements in olfactory detection and identification, reduction in qualitative distortions (e.g., parosmia or phantosmia), and enhanced quality of life.

### 1.2. Neuropsychological and Functional Consequences: Targets for Assessment and Integrated Care

Olfactory loss extends well beyond chemosensory perception, with downstream effects on everyday functioning and mental health that are highly relevant for neuropsychological practice (Table 2). Changes in appetite and eating behaviour, reduced enjoyment of food, and unintended weight loss or gain can emerge alongside safety risks (e.g., impaired detection of smoke, gas leaks, or spoiled food). In parallel, patients may experience heightened distress, reduced quality of life, and symptoms of anxiety or depression, often compounded by social withdrawal and diminished hedonic experience. Importantly, altered olfaction can also interact with cognitive functioning—through attentional demands, fatigue, and reduced environmental cueing—making it essential to include targeted screening and follow-up. An integrated assessment framework that combines chemosensory evaluation with neuropsychological and functional measures can therefore guide personalized counselling, rehabilitation priorities, and multidisciplinary care.

## 2. Clinical Vignette and Supporting Evidence

### 2.1. Patient and Baseline Assessment

MF, a 70-year-old right-handed woman with 15 years of education and no prior history of neurological or psychiatric disorders. In March 2023, she experienced an accidental fall down a flight of stairs, striking the occipital region of her head. Upon admission to the Emergency Department, she presented with an intense headache, cervical pain, nausea, and a fully preserved state of consciousness, reporting a clear recollection of the event. Clinical documentation describes stable vital signs, a Glasgow Coma Scale score of 15 [61,62], with no evidence of neurological deficits. A head CT scan performed shortly after the trauma revealed no acute intracranial lesions, haemorrhagic complications, or skull fractures, and she was discharged with a diagnosis of occipital contusive head trauma, with instructions for home monitoring and analgesic treatment.

Over the next few weeks, MF complains of significant loss of olfactory function, clinically consistent with marked hyposmia bordering on near-complete functional anosmia. Initially, she was unable to perceive even strong odours, including household cleaning products or cooking smells, and this deficit persisted without improvement for approximately four to five months. She also reported a transient disturbance in taste, as well as a mild and nonspecific sense of physical fatigue, although no other neurological complaints emerged. Because the olfactory loss remained stable over time, she sought evaluation at our centre, where a comprehensive neuropsychological assessment was performed. As part of her clinical evaluation and subsequent neuromodulation program, MF underwent a comprehensive assessment of cognitive, emotional, and quality-of-life outcomes at two time points: baseline (T0) and after completion of the neuromodulation cycle (T36). To minimize possible practice effects at follow-up, parallel or alternate versions of tests were used whenever available. A broad neuropsychological battery had been administered to evaluate global cognition, executive control, processing speed, attention, and emotional well-being (domains particularly sensitive to normal aging and to potential prefrontal dysfunction). Global cognitive efficiency had been measured with the Addenbrooke’s Cognitive Examination–Revised (ACE-R) [63], which includes the Mini-Mental State Examination (MMSE) subscore [64]. The MMSE is a widely used screening instrument for global cognitive status, with scores below 23.8 commonly considered indicative of cognitive impairment in the Italian population. The ACE-R provides a more detailed assessment across multiple cognitive domains; scores below 79 are generally regarded as suggestive of pathological cognitive decline in people under 75 years old [64]. Executive functioning and cognitive flexibility had been assessed through the Trail Making Test (TMT), administered in its parallel forms (A and C), allowing both absolute completion times and the B/A ratio to be examined [65]. Cognitive reserve had been estimated using the Cognitive Reserve Index Questionnaire (CRIq), which provided an index of MF’s lifelong intellectual enrichment and psychosocial engagement [66]. Emotional functioning had been investigated using standard self-report questionnaires: The Beck Depression Inventory–II (BDI-II) [67], the Beck Anxiety Inventory (BAI) [68], and the Fatigue Assessment Scale (FAS) [69]. Quality-of-life and perceived health had been further characterized through the EuroQol 5-Dimension, 5-Level scale (EQ-5D-5L), which allowed both a profile score and a health index to be derived [70,71]. Her baseline profile revealed normal global cognition as measured by the MMSE (26) and the ACE-R (82.4), with preserved attention, executive functioning, memory, and visuospatial abilities. Emotional functioning was likewise within typical ranges, with mild levels of self-reported fatigue (FAS: 18), minimal depressive symptoms (BDI-II: 5), and mild anxiety (BAI: 3).

MF expressed interest in participating in a neuromodulation program primarily to support cognitive well-being and prevention. However, she also reported that the persistent loss of smell had become increasingly frustrating and limiting in her daily life. She provided written informed consent for treatment and for publication of anonymized clinical information. At the time of enrolment, she was not undergoing any other treatment that might influence olfactory function.

### 2.2. Neuromodulation Protocol and Monitoring

#### 2.2.1. Cortical Excitability Assessment

Cortical excitability (CE) had been evaluated before and after the treatment program by recording motor-evoked potentials (MEPs) elicited through single-pulse transcranial magnetic stimulation (spTMS) of the left primary motor cortex (M1). A figure-of-eight coil connected to a Magstim BiStim^2^ stimulator (Magstim Company, Whitland, UK) had been used to induce a posterior–anterior monophasic current. MF had been seated comfortably with her arm supported to reduce muscular activation.

The optimal stimulation point (“motor hotspot”) had been identified as the scalp location producing the most reliable MEPs in the right first dorsal interosseous (FDI) muscle. Surface EMG had been recorded through a Biopac MP-160 system (Biopac, Goleta, CA, USA) using a belly–tendon montage, band-pass filtered (30–500 Hz), and sampled at 2 kHz. All trials with excessive background EMG or artifacts had been excluded after visual inspection, ensuring a minimum of thirty valid MEPs per time point.

The resting motor threshold (rMT) had been defined as the lowest intensity capable of producing MEPs ≥50 μV in at least 5 out of 10 consecutive stimulations [24,27,72,73,74,75]. MEPs for excitability analysis had then been recorded at 120% of the individual rMT, and peak-to-peak amplitudes had been averaged across trials [24,72,74,75]. These indices were used to characterize corticospinal excitability changes potentially associated with the neuromodulation protocol.

#### 2.2.2. rTMS Protocol

MF underwent a 12-week course of high-frequency repetitive transcranial magnetic stimulation (rTMS) targeting the left dorsolateral prefrontal cortex (DLPFC). Stimulation parameters followed an established clinical protocol already applied in our ongoing work on cortical excitability and cognitive resilience in aging [38]. The stimulation target was identified through the neuronavigation SofTaxic Navigator system (version 3.0, Electro Medical Systems, Bologna, Italy), using standardized Talairach coordinates corresponding to the left DLPFC (x = −50, y = 30, z = 36). Coil placement was maintained consistently across sessions through neuronavigation-guided marking and repositioning procedures.

Each treatment session consisted of 800 pulses delivered at 10 Hz, organized into twenty trains of forty pulses each, separated by 50-s intertrain intervals [36,37,38,76,77,78]. Stimulation intensity was set at 120% of the patient’s resting motor threshold, which was reassessed regularly to ensure accuracy [72,73,74,75,79,80,81,82,83,84,85,86]. A figure-of-eight coil connected to an STM 9000 stimulator (ATES MEDICA Device, Verona, Italy) was used for the entire protocol. Sessions were carried out thrice a week on alternate days, with each session lasting approximately seventeen minutes. Over the twelve-week period, the patient completed all thirty-six sessions without interruption.

She did not receive any concurrent medical or rehabilitative interventions that might have influenced sensory or cognitive outcomes, allowing a clear description of changes occurring during the neuromodulation period. Throughout the course of treatment, MF reported only mild and transient sensations during the initial sessions, including brief local scalp discomfort and minimal facial muscle twitching, rated 1/4 on the TMSens_Q scale [87]. These sensations resolved spontaneously without intervention.

All procedures adhered strictly to the safety and ethical recommendations of the International Federation of Clinical Neurophysiology (IFCN).

### 2.3. Outcomes

A structured diary was used to record changes in olfactory perceptual capacity during the treatment. Standardized psychophysical tests for smell were not available at the start of treatment, but the diary offered a way to monitor changes throughout the intervention, giving a continuous and realistic record of how MF’s sense of smell changed. In addition to the longitudinal odour diary, subjective olfactory functioning and its impact on daily life were assessed before and after the neuromodulation treatment using self-report questionnaires, including the Self-reported Mini Olfactory Questionnaire (Self-MOQ) [88] and the Italian brief version of the Questionnaire of Olfactory Disorders (Brief-IT-QOD) [89]. The Self-MOQ indexed the perceived severity of olfactory loss, while the Brief-IT-QOD characterised the quality-of-life burden associated with olfactory dysfunction, distinguishing between parosmia-related complaints (QOD-P) and broader negative statements reflecting daily-life impact (QOD-NS). These instruments captured MF’s subjective experience of olfactory dysfunction rather than objective sensory performance. Baseline and post-treatment questionnaire scores are reported in Table 3.

During the early sessions, MF did not notice substantial improvement. However, from around the tenth to twelfth therapy sessions, she gradually began noticing strong and pungent smells that had been completely missing since the trauma. These first olfactory perceptions began with the sense of burning, smoking, and chemical odours from cigarettes. Despite their inconsistency, these early sensations represented the first signs of recovery after months of complete hyposmia.

As treatment progressed into the mid-phase, around sessions twelve to twenty-four, MF noticed more stable and reliable smells. She started to recognize the smell of coffee, cooked food, citrus peel, and personal perfumes. These smells, previously experienced in vague, intermittent episodes, gradually became stronger and easier to identify in the weeks to come. Additionally, she noted that odours previously detectable only in close proximity began to be detectable from a distance in a room. Towards the final third of the treatment phase, between sessions twenty-four and thirty-six, MF reported a marked qualitative change in her experience of smell. She began to experience fine and subtle scents that other people in the area perceived as barely detectable. Finally, she described her sense of smell as richer and more complex, and felt she had returned to her normal sensitivity.

**Figure 2:**
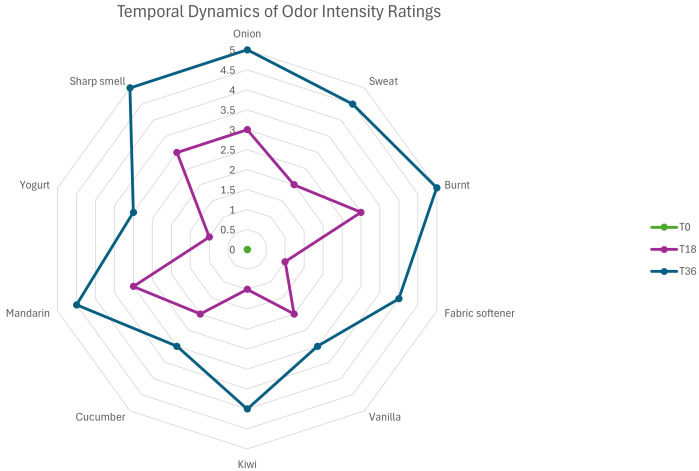
**Longitudinal** **changes in self-reported olfactory perception across the rTMS intervention.** Radar plot showing odor-intensity ratings for ten odor categories (onion, sweat, burnt, fabric softener, vanilla, kiwi, cucumber, mandarin, yogurt, and sharp chemical smell) at baseline (T0), mid-treatment (T18), and post-treatment (T36). Odor intensity was rated on a Likert-type scale ranging from 0 (no perception) to 5 (very strong odor). At baseline, ratings were near zero across all odor categories, consistent with global hyposmia. At mid-treatment, increased ratings emerged selectively for strong or pungent odors (e.g., burnt, sweat, sharp chemical smells). At post-treatment, higher ratings were observed across all odor categories, including more subtle scents.

Figure 2 shows a radar plot that illustrates how MF’s sense of smell changed over time. The plot displays ratings for ten types of odours, including onion, sweat, burnt, fabric softener, vanilla, kiwi, cucumber, mandarin, yogurt, and sharp chemical smells, at three different times: T0, T18, and T36. Odor intensity was rated on a Likert scale from 0, meaning “no perception,” to 5, meaning “very strong odour.” At T0, her ratings were almost all zero, matching her report of hyposmia. At T18, she started to rate strong or sharp smells, like burnt, sweat, and chemical scents, as more intense, which matched her first signs of improvement. By T36, the plot demonstrates a generalized restoration of olfactory sensitivity, with substantial increases across all odour categories, including subtle scents such as yogurt, vanilla, and cucumber, which MF reported as newly detectable during the final weeks of treatment.

**Figure 3:**
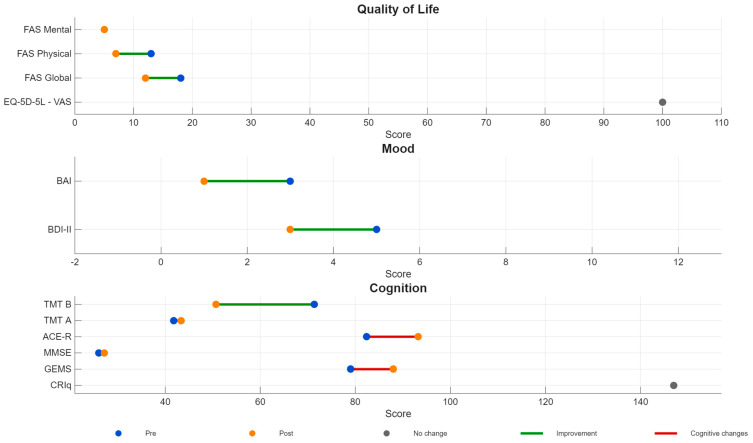
**Neuropsychological** **and emotional outcomes before and after rTMS.** The figure illustrates changes across global cognitive functioning (MMSE, ACE-R), executive performance (Trail Making Test–B completion time), emotional symptoms (BDI-II, BAI), perceived fatigue (FAS), quality of life (EQ-5D-5L), and cognitive reserve (CRIq). Where available, clinical cut-off values are indicated in the direction of normal performance. Differences between baseline and follow-up are displayed as change scores (Δ = post − pre), with positive values reflecting improvement according to the normative direction of each measure. For measures in which higher scores indicate better functioning (e.g., MMSE, ACE-R, EQ-5D-5L, CRIq), positive Δ values reflect improved performance. For measures in which lower scores or shorter completion times indicate better functioning (e.g., BDI-II, BAI, FAS, TMT-B), negative Δ values reflect improvement. Clinical cut-off values, where available, are defined according to established normative criteria and are described in the ‘Patient and baseline assessment’ section. In this context, the term ‘cognitive changes’ denotes any measurable variation in test performance across time, regardless of direction. The term ‘improvement’ refers specifically to changes occurring in the direction of better functioning relative to the normative reference for each measure. *Abbreviations*: ACE-R, Addenbrooke’s Cognitive Examination–Revised; BAI, Beck Anxiety Inventory; BDI-II, Beck Depression Inventory–II; CRIq, Cognitive Reserve Index Questionnaire; EQ-5D-5L, EuroQol 5-Dimension 5-Level; FAS, Fatigue Assessment Scale; MMSE, Mini-Mental State Examination; TMT, Trail Making Test.

After the active rTMS treatment, the neuropsychological assessment showed improvements in several areas. MF’s MMSE score went up from 26 to 27.2, and her ACE-R score rose from 82.4 to 93.2. Her executive function also improved, as shown by a faster completion time on the Trail Making Test–B, dropping from 71.4 to 50.7 s. Emotional well-being also improved, with reductions in both depressive and anxiety symptoms, and her perceived fatigue decreased meaningfully (Figure 3). No adverse effects were recorded at any time. The patient specifically reported feeling more alert and energetic and did not experience any fluctuations or worsening in olfactory perception during treatment.

**Figure 4:**
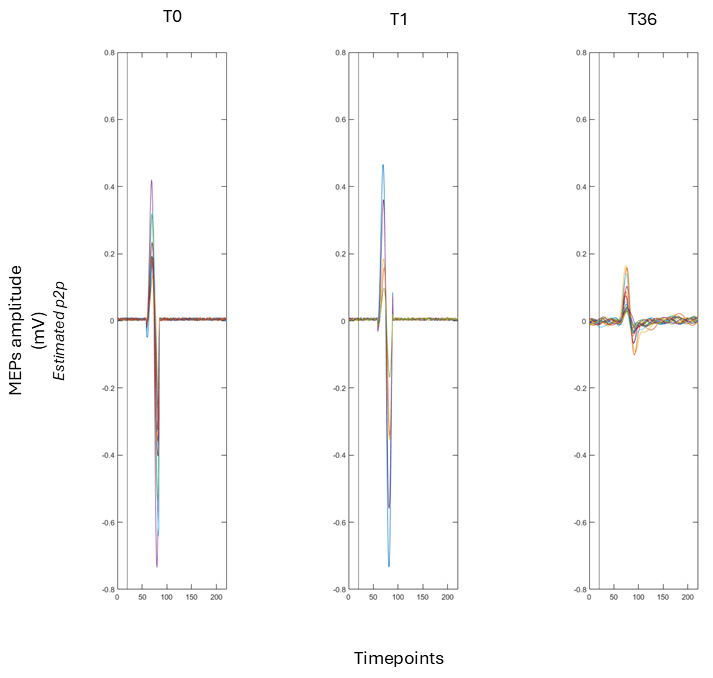
Assessment of cortical excitability measured through mean peak-to-peak MEP amplitudes (mV) at baseline (T0), mid-treatment (T1), and post-intervention (T36).

Cortical excitability data derived from motor-evoked potentials were consistent with broader clinical and cognitive changes observed during the intervention period (Figure 4). A linear model examining peak-to-peak MEP amplitudes across time points (T0, T1, T36) showed an effect of time (F(2, 43) = 28.50, *p* < 0.001). Mean MEP amplitudes decreased progressively from 0.84 mV at baseline (T0) to 0.62 mV at mid-treatment (T1) and 0.10 mV at post-treatment (T36). Post hoc Tukey comparisons confirmed reductions between T0 and T36 (Δ = −0.74 mV, *p* < 0.001) and between T1 and T36 (Δ = −0.52 mV, *p* < 0.001), indicating a marked decrease in corticospinal excitability following active rTMS. Importantly, these neurophysiological measures were not intended to capture the earliest subjective olfactory changes, which emerged around sessions 10–12, but rather to index cumulative, network-level modulation across the treatment course. In this context, the observed reduction in corticospinal excitability converged with the overall pattern of cognitive, emotional, and sensory improvement, consistent with a neuromodulatory effect on prefrontal–motor circuits.

## 3. Mechanistic Interpretation and Clinical Implications

This clinical vignette describes the gradual recovery of olfactory function in a woman with post-traumatic hyposmia, temporally associated with a course of high-frequency rTMS applied to the left DLPFC [1,2,90,91]. Although the patient’s CT scan did not reveal structural abnormalities, olfactory dysfunction after mTBI frequently occurs even when routine CT scans are unremarkable [92]. This is because microstructural injuries of the olfactory fila, olfactory bulb, or orbitofrontal pathways often escape CT resolution, despite producing significant functional impairment [2,90,92]. Shearing of olfactory axons at the cribriform plate, oedema or microhaemorrhages in the orbitofrontal cortex, and subtle disruptions in fronto-limbic connectivity are recognized mechanisms underlying post-traumatic olfactory loss even in cases with normal CT findings [2,92,93]. These forms of “silent” or non-visible injury may impair olfactory function while leaving gross neuroimaging unrevealing. The prolonged stability of the deficit prior to treatment, followed by a clearly phased recovery during rTMS, suggests that neuromodulation may have facilitated reorganization or increased responsivity within these compromised pathways. Importantly, the potential efficacy of left DLPFC rTMS should be interpreted in light of these heterogeneous underlying mechanisms. While prefrontal neuromodulation is unlikely to directly promote peripheral reafferentation or structural repair of olfactory axons in cases dominated by mechanical damage at the cribriform plate, it may exert clinically meaningful effects in patients whose olfactory dysfunction is sustained or amplified by central and network-level alterations [94,95]. In such cases, disrupted fronto-limbic connectivity, impaired top–down attentional modulation, or altered reward-related processing may contribute to the persistence of sensory deficits even in the absence of overt structural lesions [96,97]. From this perspective, the clinical impact of DLPFC-rTMS is likely to be aetiology-dependent, with greater plausibility in forms of PTOD characterised by central or connectivity-related dysfunction rather than purely peripheral injury.

The pattern of recovery observed in MF is consistent with known dynamics of olfactory reafferentation. The earliest regained sensations consisted of intense and aversive odors such as smoke and burning, which typically have higher perceptual salience and may be mediated by partly distinct neural pathways [1,2,8,90,92]. Notably, this phase of early olfactory recovery was accompanied by improvements in cognitive and emotional functioning, suggesting a potential interaction between sensory recovery and higher-order processes. Those cognitive and affective changes may have indirectly facilitated olfactory recovery by enhancing top–down attentional engagement, motivational drive, and reward sensitivity, thereby increasing the salience of olfactory inputs and supporting multisensory integration [98,99]. In this framework, olfactory improvements may reflect not only direct modulation of sensory networks, but also a more favourable cognitive–emotional context for sensory awareness and perceptual learning [98,99,100]. Accordingly, improvements in cognitive and emotional functioning and gains in olfactory perception are likely to be reciprocally related, reflecting interacting components of a broader network-level recovery process rather than independent treatment effects.

As treatment progressed, MF’s perceptual repertoire broadened to encompass both common and complex odors, ultimately resulting in the renewed ability to detect subtle fragrances. The nature of this recovery, which unfolded over the same time frame as the neuromodulation sessions, suggests a dose-dependent or cumulative effect.In addition to perceptual improvements, MF demonstrated clear gains in cognitive and emotional functioning, consistent with the established role of left-DLPFC rTMS in modulating executive control, attention, and mood regulation [21,23]. Improved Addenbrooke’s Cognitive Examination-Revised (ACE-R) scores, faster executive performance on the Trail Making Test Part B (TMT-B), and reductions in depressive and anxiety symptoms all align with the expected therapeutic profile of high-frequency prefrontal rTMS.

Physiological data provided further convergent evidence. Peak-to-peak MEP amplitudes recorded from the right FDI decreased significantly across the intervention period, indicating a reduction in corticospinal excitability. In the context of prefrontal neuromodulation, reduced corticospinal excitability should not be interpreted as a loss of functional capacity. Rather, converging evidence suggests that such changes may reflect a normalisation of baseline excitability levels, increased inhibitory control, or a rebalancing of excitatory–inhibitory interactions within fronto-striatal and prefrontal–motor circuits [94,101]. From this perspective, decreased MEP amplitudes may indicate a transition toward a more efficient and regulated network state, consistent with improvements in executive functioning, attentional stability, and emotional regulation observed at the behavioural level [94,101]. Although the stimulation target was prefrontal rather than motor, DLPFC stimulation is known to exert downstream effects on motor cortex excitability through fronto-striatal and transcallosal networks [21,23,45]. The progressive reduction in MEP amplitude observed from baseline (T0) to the mid-intervention assessment (T1) and further to the post-treatment assessment (T36) is consistent with the literature showing that prefrontal neuromodulation can influence the intracortical inhibition–facilitation balance within the primary motor cortex (M1). Notably, the temporal evolution of corticospinal excitability occurred alongside the progressive clinical and behavioural changes observed during the rTMS course, supporting a network-level interpretation in which prefrontal stimulation is associated with coordinated changes across sensory-perceptual, cognitive, and motor-related systems. Within this framework, MEP modulation can be considered an indirect physiological index of stimulation-related network reorganisation.

Standardized olfactory psychophysical testing (e.g., Sniffin’ Sticks) was not available at the time of evaluation. In line with previous clinical guidelines, we relied on a structured odour diary, a method widely used in clinical otorhinolaryngology when longitudinal monitoring is required and formal testing cannot be performed [102,103]. Diary-based assessments provide high ecological validity and are particularly informative in cases with progressive intra-individual recovery [90]. However, it should be acknowledged that diary-based and self-report approaches do not allow independent assessment of specific psychophysical components of olfaction, such as detection threshold, odour discrimination, or odour identification. Consequently, our observations cannot disentangle which perceptual dimensions primarily contributed to recovery, limiting fine-grained mechanistic interpretation. Future studies should therefore combine standardised psychophysical testing with ecological diary-based monitoring to capture both component-level sensory changes and real-world subjective experience.

Alternative explanations must be considered, including the possibility of spontaneous recovery. However, the patient showed a stable plateau for approximately five months before entering the rTMS program, with no spontaneous improvement. The first signs of olfactory recovery emerged only after approximately 10–12 sessions, following a gradual, dose-dependent trajectory consistent with stimulation timing rather than spontaneous remission. Placebo effects cannot be excluded but seem insufficient to account for the structured, stepwise pattern of sensory recovery she reported [2,103,104]. While causality cannot be established from a single case, the temporal coupling between stimulation and improvement provides a compelling rationale for further study [102,103].

Overall, this case supports the hypothesis that high-frequency rTMS over the DLPFC may modulate olfactory function indirectly by enhancing the plasticity of frontal networks that regulate multisensory integration and top–down attention to sensory stimuli. These findings contribute to a growing body of literature suggesting that neuromodulatory interventions may have wider-ranging effects than traditionally assumed, extending beyond cognitive and affective domains into the realm of sensory processing.

## 4. Research Agenda and Future Directions

The observation reported here should be treated as hypothesis-generating. To move the field forward, future work on PTOD and neuromodulation should prioritize rigorous outcome measures, mechanistic readouts, and designs that can separate stimulation effects from spontaneous recovery and expectancy.

We propose the following practical priorities for studies testing prefrontal rTMS as an adjunct or standalone intervention for olfactory dysfunction:

1. Use standardized psychophysical olfactory testing (e.g., threshold, discrimination, identification) at baseline and follow-up, complemented by ecologically valid diaries for within-person trajectories.
2. Adopt randomized, sham-controlled designs (or at minimum multiple-baseline single-case designs) and report expectancy and blinding integrity.
3. Specify and justify targeting (neuronavigation when possible) and systematically explore dose parameters (frequency, intensity, total pulses, number of sessions) and laterality.
4. Evaluate combinations with evidence-informed olfactory training and/or hedonic stimulation to leverage attention and reward mechanisms in multisensory recovery.
5. Add mechanistic biomarkers (EEG, fMRI, PET, connectivity measures, or TMS-EMG indices) to test network-level hypotheses and identify responders.
6. Report safety and tolerability in older adults and in patients with head trauma, including adverse event monitoring and follow-up durability of gains.

## 5. Conclusions

Although direct evidence supporting rTMS for olfactory dysfunction remains limited, the clinical vignette contributes to the field by illustrating how prefrontal neuromodulation may be explored within a network-based framework in post-traumatic olfactory dysfunction. The case is distinguished by its post-traumatic aetiology, the longitudinal and session-by-session tracking of olfactory experience across a full rTMS course, the integration of sensory, cognitive, emotional, and cortical excitability measures within a single individual, and the use of a prolonged and clinically realistic stimulation protocol. Together, these elements allow a fine-grained observation of temporal dynamics and cumulative effects that are often inaccessible in shorter or purely pre–post designs. While causality cannot be inferred from a single case, our “perspective article” highlights key methodological and mechanistic considerations for future controlled studies and supports the rationale for investigating prefrontal rTMS as a hypothesis-generating, network-level approach in selected cases of post-traumatic olfactory dysfunction.

## Figures and Tables

**Table 1:** Therapeutic options for acquired olfactory dysfunction: evidence snapshot and typical clinical contexts. Reference articles are marked in bold in the full references.

*Intervention*	Rationale/Mechanism	Typical Protocol (Examples)	Evidence Snapshot
*Olfactory training (OT)* *(classic/modified/intensive) [47,48,49]*	Peripheral + central plasticity; top-down attentional engagement; repeated odor exposure	4 odors, 2×/day, ≥12 weeks; extended protocols (6–9 months); intensive variants in persistent post-COVID	Highest level of evidence across post-viral OD; recommended first-line. Benefits may increase when combined with adjuncts.
*Corticosteroids* *(topical or systemic; selected cases) [50,51,52]*	Anti-inflammatory effects; may help when sinonasal inflammation present or early post-viral phase	Short course systemic steroids or topical sprays; usually combined with OT	Evidence mixed/heterogeneous; commonly used but optimal indications unclear; risk–benefit individualized.
*Platelet-rich plasma (PRP)* *(intranasal/olfactory cleft injection) [53,54]*	Pro-regenerative growth factors; anti-inflammatory; supports epithelial and neural repair	1 mL per cleft, often repeated; protocols vary (injection vs. topical carriers)	Promising results in post-viral/post-COVID OD; growing evidence base, including meta-analyses; several trials ongoing.
*Adjunct nutraceuticals* *(e.g., PEA-luteolin; omega-3, etc.) [55]*	Anti-inflammatory and neuroprotective pathways; modulation of glial activation (hypothesized)	Typically combined with OT; dosing varies by product and study	Preliminary evidence suggests OT + adjuncts may improve recovery vs. OT alone, but standardization and replication needed.
*Neuromodulation: tDCS + OT [56,57]*	Modulation of network excitability and plasticity; may enhance OT-driven learning	Anodal tDCS paired with OT (double-blind protocols reported)	RCT evidence emerging in persistent post-COVID anosmia; effect sizes and durability still being defined.
*Neuromodulation: rTMS* *(prefrontal targets; case-based evidence) [58]*	Top-down control of olfactory–limbic networks; dopaminergic/reward and attentional systems (hypothesized)	High-frequency rTMS over left DLPFC (protocols vary)	Sparse direct evidence for OD; case reports/case series suggest potential benefit; mechanistic rationale motivates trials.
*Other pharmacologic/non-pharmacologic options* *(e.g., vitamin A, sodium citrate, insulin, theophylline, acupuncture) [47,59]*	Heterogeneous mechanisms (epithelial regeneration; receptor modulation; neurometabolic effects)	Varies widely	Generally low-to-moderate evidence with heterogeneity; may be considered experimental or context-dependent.

**Note**: The level of evidence supporting neuromodulation-based interventions varies across the different causes of olfactory dysfunction. To date, stronger and more systematic evidence is available for post-viral (including post-COVID-19) olfactory dysfunction, while evidence for post-traumatic olfactory dysfunction remains limited and is largely based on case reports.

**Table 2:** Neuropsychological and functional consequences of olfactory dysfunction: suggested assessment targets and clinical implications. Reference articles are marked in bold in the full references.

*Domain*	Key Findings (Summary)	Suggested Assessment Targets	Clinical Implications
*Olfactory-specific quality of life [60]*	Olfactory loss impacts daily life (eating, social, hazards) and can be severe for a subset of patients.	QOD (Questionnaire of Olfactory Disorders), visual analogue scales; patient diary	Track treatment benefit beyond psychophysics; identify domains needing rehabilitation and counseling.
*Mood and distress (depression/anxiety) [60]*	Persistent OD is associated with higher depression/anxiety and distress, particularly post-COVID.	HADS/PHQ-9/GAD-7; clinical interview	Screen routinely; consider combined sensory rehabilitation + psychological support; monitor anhedonia.
*Cognition (attention, executive function, memory) [60]*	Associations between olfaction and cognition reported across populations; interventional evidence limited.	Global cognitive screen + domain tests (e.g., ACE-R/MMSE; TMT; verbal memory)	Use cognitive profiling to tailor interventions; investigate whether sensory recovery co-varies with cognition.
*Eating behavior, nutrition and weight [60]*	OD can alter food enjoyment, appetite, dietary choices and may contribute to weight change.	Dietary history; weight/BMI; eating behavior questionnaires (as available)	Provide dietary counseling and safety guidance; monitor involuntary weight loss.
*Safety and hazard detection [60]*	Reduced ability to detect smoke, gas leaks and spoiled food increases environmental risks.	Structured safety checklist; caregiver report	Implement compensatory strategies (alarms, labels, routines); provide written safety advice.
*Social and hedonic functioning [60]*	Olfaction contributes to social communication and hedonic experience; OD may reduce social engagement.	Patient-reported outcomes; social functioning scales (as available)	Psychoeducation; address avoidance and social withdrawal; consider partner/family counseling.

**Table 3:** Self-reported olfactory outcomes before and after rTMS: Subjective olfactory dysfunction severity and olfactory-related quality of life were assessed at baseline (T0) and after completion of the rTMS programme (T36) using validated self-report questionnaires.

*Measure*	Score Range	Baseline (T0)	Post-Treatment (T36)	Direction of Improvement
*Self-reported Mini Olfactory Questionnaire * * (Self-MOQ) [88]*	0–5	5	1	↓ Lower scores indicate better perceived olfactory function
*Brief-IT-QOD—QOD-P * * (Parosmia) [89]*	0–12	1	0	↓ lower = fewer parosmia-related complaints
*Brief-IT-QOD—QOD-NS (QoL burden)*	0–21	14	4	↓ lower = reduced olfactory-related QoL burden
*Brief-IT-QOD—Total*	15	15	4	↓ lower = reduced overall burden

## Data Availability

The raw data supporting the conclusions of this article will be made available by the authors, without undue reservation.

## Abbreviations

The following abbreviations are used in this manuscript:

ACE-R	Addenbrooke’s Cognitive Examination–Revised
BAI	Beck Anxiety Inventory
BDI-II	Beck Depression Inventory–II
CE	cortical excitability
CRIq	Cognitive Reserve Index questionnaire
CT	computed tomography
DLPFC	dorsolateral prefrontal cortex
EQ-5D-5L	EuroQol 5-Dimension 5-Level
EMG	electromyography
FAS	Fatigue Assessment Scale
FDI	first dorsal interosseous
GAD-7	Generalized Anxiety Disorder-7
GCS	Glasgow Coma Scale
HADS	Hospital Anxiety and Depression Scale
MEP	motor-evoked potential
M1	primary motor cortex
MMSE	Mini-Mental State Examination
OD	olfactory dysfunction
OFC	orbitofrontal cortex
OT	olfactory training
PHQ-9	Patient Health Questionnaire-9
PTOD	post-traumatic olfactory dysfunction
QOD	Questionnaire of Olfactory Disorders
QOD-NS	negative statements subscale of the Questionnaire of Olfactory Disorders
QOD-P	parosmia subscale of the Questionnaire of Olfactory Disorders
rMT	resting motor threshold
rTMS	repetitive transcranial magnetic stimulation
Self-MOQ	Self-reported Mini Olfactory Questionnaire
spTMS	single-pulse transcranial magnetic stimulation
TMT	Trail Making Test
T0	baseline
T36	post-treatment
